# Collision-based synthesis of diamond–graphite nanocomposites: computational investigation

**DOI:** 10.1039/d6ra04047k

**Published:** 2026-07-20

**Authors:** Zuzanna Malinowska-Trzmielak, Nicole Grobert, Mark Wilson

**Affiliations:** a Physical and Theoretical Chemistry Laboratory, Department of Chemistry, University of Oxford UK zuzanna.trzmielak@chem.ox.ac.uk; b Department of Materials, University of Oxford UK nicole.grobert@materials.ox.ac.uk; c Department of Chemistry, University of Oxford UK mark.wilson@chem.ox.ac.uk

## Abstract

Diamond–graphite nanocomposites have attracted significant attention due to their unusual structure, combining graphitic and diamond domains within a single material, as well as their promising electronic and mechanical properties. However, existing synthesis routes typically rely on poorly controlled high-pressure, high-temperature phase transitions, limiting the ability to “tune” the relative proportions of graphite and diamond. Here, we explored an alternative strategy that avoids bulk phase transformations and instead targets the formation of interfaces between pre-existing graphite and diamond domains under shock conditions, such as those generated by high-speed collisions. Using molecular dynamics simulations of simplified particle–particle impacts, we assessed the feasibility of collision-driven interfacial bonding under idealised conditions across multiple diamond facets and graphite orientations. In principle, collisions should generate localised interfacial heating that promotes bond formation without necessarily disrupting the internal structure of either domain. For hydrogen-terminated surfaces, the average relative collision velocity that should result in formation of a coherent interface was estimated to be approximately 2500 m s^−1^, with lower values expected upon appropriate surface functionalisation. At higher velocities, graphitic layers undergo significant deformation and transition toward a diamond-like amorphous phase, with thresholds of approximately 3246 m s^−1^ for [0001]_G_ and 3810 m s^−1^ for [101̄0]_G_, in good agreement with prior studies. Overall, this work provides a theoretical argument for an alternative diaphite synthesis route based on controlled interfacial reactions rather than bulk phase changes. Such an approach may enable improved control over domain sizes and phase composition. Synthesis techniques such as thermal spraying, spark plasma sintering, and explosive powder compaction may prove useful in its implementation.

## Abstract 2

Molecular dynamics simulations identify collision-induced interfacial bonding as a feasible alternative to conventional high-pressure synthesis of diamond–graphite nanocomposites, offering improved control over structure and composition.

## Graphical abstract

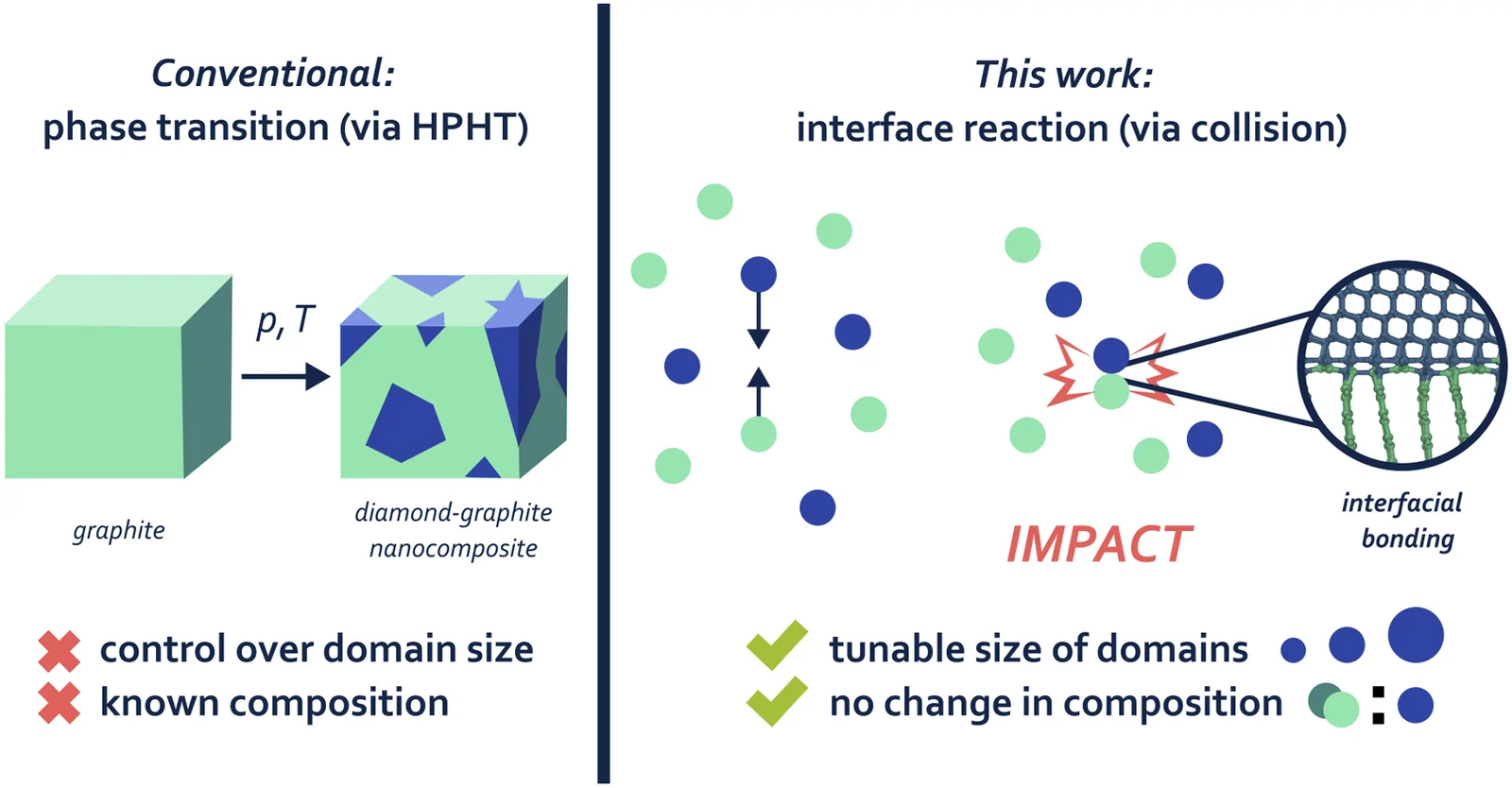

## 1 Introduction

Diaphites, or diamond–graphite nanocomposites, are a novel form of carbon first discovered in meteorite impact samples.^1^ Similar to amorphous carbon, they feature sp^2^- and sp^3^-hybridised carbon atoms. However, in diaphites, these atoms are arranged in single-phase domains connected by a coherent grain boundary, referred to as gradia.^2^ Owing to the preserved crystallinity within individual domains, they are predicted to display a range of unique properties that combine the contrasting qualities of graphite and diamond or mitigate the limitations of each. Ge *et al.* predict high-temperature superconductivity due to the phonon–electron junction at the grain boundary.^3^ Other studies highlight improvements in mechanical properties. Neméth *et al.* point out that the graphitic domain embedded in a diamond matrix could act as a crack-energy dissipator by redirecting fracture energy towards graphitization.^4^ Li *et al.* demonstrated that gradia may play an important role in extreme elongation under tensile stress due to step-wise transformation of diamond domains rather than specimen failure.^5^ These behaviours are not observed in graphite or diamond individually and, as a result, diaphites have attracted significant attention in recent years.

All of these properties relies on the presence of two distinct types of domains. Since the properties of other carbon-based materials are heavily influenced by the sp^2^/sp^3^ ratio,^6^ it is likely that the same applies to diaphites to some extent. Therefore, to reach their full potential, the synthetic route must allow for effective control over the diamond-to-graphite ratio. Unfortunately, the high-pressure, high-temperature (HPHT) method, which is currently the most established route toward diaphite synthesis, falls short in this regard. It involves subjecting graphite to extreme conditions and inducing a partial phase transition to form domains.^2,7^ However, the relationship between the reaction conditions and the structural outcomes remain unclear. Although many mechanisms have been proposed,^2,8,9^ they aim to explain how graphite transforms into diamond but not how it remains as a mixed-phase diaphite. Consequently, no conclusions can be drawn on how to control the structure of the final product, including the sp^2^/sp^3^ ratio. The method therefore remains highly empirical, leaving the outcome unpredictable, as suggested by reports subjecting graphite to very similar HPHT conditions but obtaining diamond instead.^10,11^ These issues, combined with the high costs and scale-up limitations associated with low production volume, indicate that HPHT method might not be the most suitable route for controlled synthesis of diaphites, and that alternative pathways should be explored.

Although Zhai *et al.* reported diaphite formation under microwave plasma-enhanced chemical vapour deposition,^12^ this process suffers from similar limitations regarding outcome control. Moreover, like the HPHT method, it is derived from industrial diamond-synthesis techniques.^13^ Both approaches rely on modifying an existing diamond-production method in the hope of obtaining diaphite. Although this strategy has yielded results, it may not be the most effective way forward, as it requires a delicate balance between thermodynamically stable and metastable states. As a result, diaphite synthesis may require a novel and dedicated approach.

To design such an approach, the first step is to shift the current perspective, which tends to treat diaphites as a singular entity. The literature emphasises the connection between domains—understandably so, as the interface is what distinguishes diaphites from other carbon materials. However, this perspective may not be the most useful for synthesis design. A more practical viewpoint is to treat the diamond–graphite nanocomposite as a solid mixture of two polymorphs connected by a grain boundary. Because both graphite and diamond are readily available, in principle, a conceptual route to diaphite formation would be to connect the surfaces of these two phases.

Creating the grain boundary would require breaking bonds at the interface. Depending on the diamond surface and/or graphitic edge, this might involve breaking C–H, C–O or C–C bonds.^14–17^ Bond breaking is an energy-consuming process. Although the HPHT method, as a high-energy process, could facilitate such reactions, replacing graphite as the sole starting material with a mixture of diamond and graphite would be impractical. First, it would increase the cost of the starting material. Second, the extreme conditions would still allow the proportion of the polymorphs to change significantly. The energy barrier associated with the phase transition would easily be overcome, leading to a change in the initial diamond-to-graphite ratio. Instead, we propose subjecting diamond and graphite to shock conditions, namely high-speed collisions. The kinetic energy converted to heat at the interface could be sufficient to facilitate bond breaking. At the same time, due to the brief nature of the collision, the system would not have enough energy input to undergo a phase transition, unless the kinetic energy reached a threshold at which point, structural changes become inevitable.

This work explores the hypothetical synthesis of diaphites based on high-speed collisions using molecular dynamics simulations. It analyses structural changes as a function of impact strength and relates them to the diamond facet involved in the collision. It provides an approximate collision speed that is likely to result in coherent interface formation between diamond and graphite particles. It also estimates the critical speed leading to significant deformation and structural changes and compares these findings with existing shock studies. Lastly, it briefly considers the available synthetic techniques that could move these predictions from theory to practice.

## 2 Methods

**Fig. 1:**
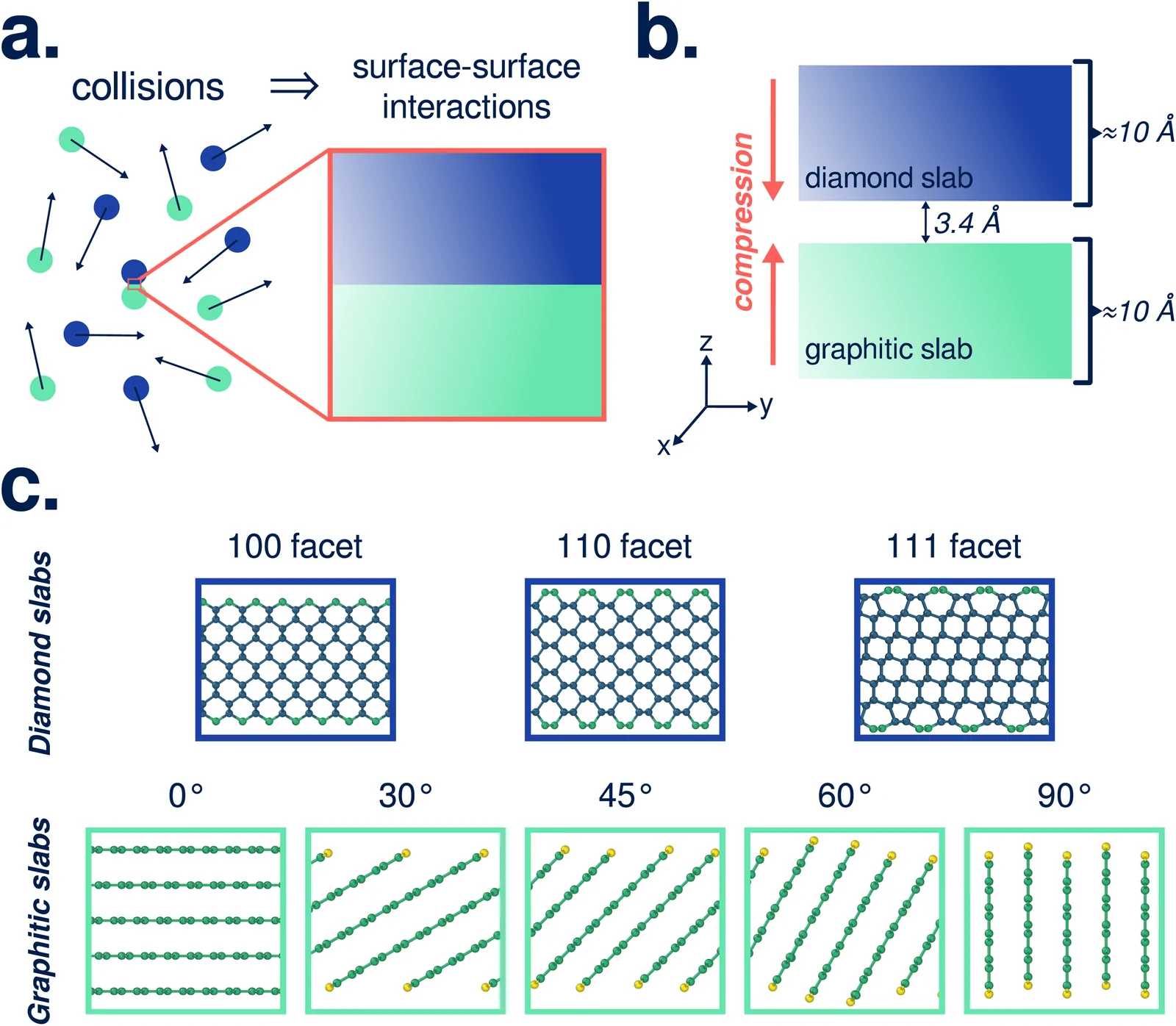
(a) Representation of collisions on macro and atomic scales, where collision between two particles can be approximated to contact between two surfaces. (b) Schematic representation of the starting system with diamond and graphitic slabs to be compressed anisotropically in the *z* direction of the super cell. (c) Diamond and graphitic slabs included in the simulations, leading to 15 different systems in total. Blue, green and yellow atoms refer to four, three and two-coordinated atoms.

Simulating collisions is an inherently complex problem. However, on the atomic level collision can be approximated to two material surfaces coming together and interacting (Fig. 1a). This approximation was adopted throughout the study.

The starting configuration consisted of two slabs, diamond and graphitic, approximately 10 Å thick, so two surfaces of a single slab can be treated as independent, similarly to bulk crystal. The slabs were separated by 3.4 Å, which corresponds to minimal, non-interacting distance, based on the sum of van der Waals radii.^18^ (Fig. 1b) The slabs were forced into contact by controlled cell anisotropic deformation in the *z* direction with respect to the slabs (defined as in Fig. 1), applied over 25 ps. The deformation varied from 5 to 45% compression with respect to the initial cell length in the *z* direction. It was performed with controlled volume and temperature, under *NVT* ensemble, where *T* = 300 K and damping constant *τ*_temp_ = 100 ps. The damping constant was explicitly set to a relatively high value to allow for a largely unrestricted rise in temperature, yet reflecting the experimental conditions which usually occur at fixed temperature. The cell was then relaxed with controlled pressure and temperature, under *NPT* ensemble, over 100 ps, where *T* = 300 K, *p* = 0 bar, and their damping constants were equal to *τ*_temp_ = 10 ps, *τ*_pressure_ = 100 ps, respectively. Long relaxation times for thermostat and barostat were used to avoid over-damping the temperature spike characteristic of shock events. All MD simulations were performed in LAMMPS^19^ with the ACE potential.^20^ This choice was dictated by benchmark study against *ab initio* Molecular Dynamics (PBE + D2).^21^ ACE potential was determined to give the most accurate description for simulations involving diaphites, including sp^2^–sp^3^ carbon interconversion and associated electronic changes which are implicitly included within the potential parameters. Simulations employed an integration time step of 1 fs and periodic boundary conditions (PBC). PBC was deemed appropriate since the upper and lower surfaces were equivalent and the periodic image of another slab acted essentially like the rest of the bulk that would normally be present in experiment.

Three distinct diamond slabs were constructed, each cut along different facets (100, 110 and 111), all C-terminated (Fig. 1). Graphitic slabs were varied by setting the [101̄0]_G_ direction orientation with respect to the diamond surface to 0°, 30°, 45°, 60° and 90° (Fig. 1c). Graphitic layers were C-terminated with the zigzag edge on both sides, due to the lower energy compared to the armchair edge.^14^ Presence of heteroatoms was accounted for in the speed of collisions calculations outlined in Section 3.2. This omission was, somewhat counter intuitively, dictated by the need for accurate representation of nanocomposite structure in the simulation. None of the multi-species MD potentials was neither trained nor benchmarked for diaphites. Those that were benchmarked for diaphites are carbon-only potentials. Therefore, we opted in for the solution of known accuracy at the expense of simplified description of the system. Each diamond surface was paired with each orientation of the graphitic planes, leading to 15 different starting configurations. The supercells were visualised with OVITO.^22^

## 3 Results and discussion

### 3.1 Simulations

Result analysis involved looking at the structural changes exerted on the slabs and relating them to changes in cell length in the *z* direction, temperature, and pressure over time. Due to the employed simplifications, like lack of heteroatoms at the surfaces/edges, the speed of collisions had to be estimated based on changes in those. Detailed methodology can be found in Section 3.2.

**Fig. 2:**
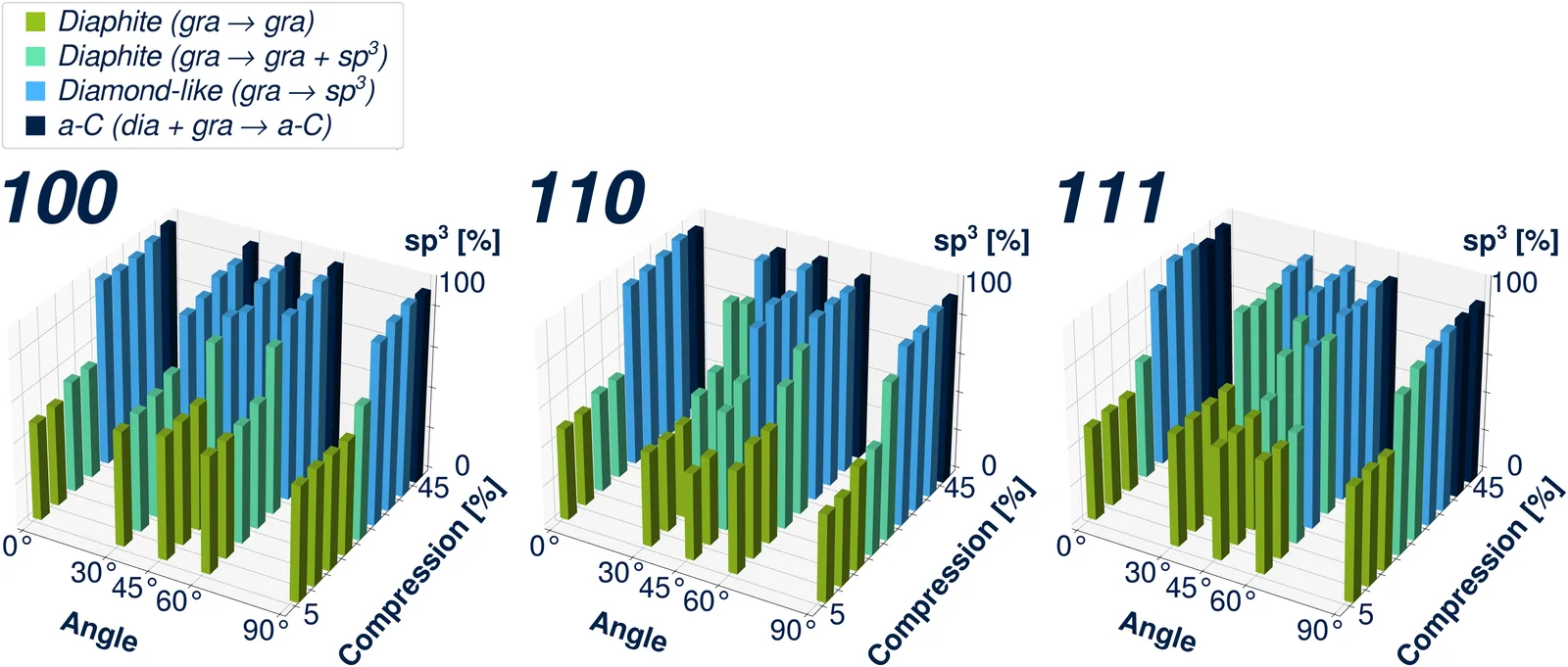
The final sp^3^ percentage in obtained structures with respect to diamond facet, orientation angle of graphitic layers and introduced compression. Colours indicate type of the structure and transformations within domains and were assigned based on change in percentage of cubic diamond estimated with common neighbour analysis^23^ as implemented in OVITO, along with change in percentage of 3-coordinated atoms, supplemented by visual inspection. The decision making tree is presented in the SI and the calculated percentages can be found in the database associated with this work.

According to Fig. 2, all simulations resulted in bonding between graphite and diamond slabs, leading to various structures depending on compression. However, it is not evidence in its own right due to unrealistic, yet useful, for the simulation purposes, graphitic edge and diamond surface representations. The advocation for the plausibility of the bonding must be derived from other means – those that bridge the gap between the model and are related to the conditions of collisions.

An important part of the collision event is localised heating at the interface – essential in facilitating bond breaking and forming. This is especially important when considering the relatively high bond dissociation energy of C–H (≈420 kJ mol^−1^) and C–O (≈385 kJ mol^−1^) bonds,^24^ commonly found at the surfaces in question. Therefore, the temperature at the interface needs to be quite high, likely similar to that used in thermal cracking on alkenes, namely *T* ≈ 1000 to 1300 K.^25^ Although not identical, both involve C–H bond cleavage, and similar analogies between simple organic species and H-terminated carbon surfaces have been made previously.^26^ Temperature as a thermodynamic, static quantity can be related to the kinetic energy of particles. This in turn is related to their velocities, giving rise to the following relationship:*T* ∝ *E*_k_*T* ∝ *v*^2^

**Fig. 3:**
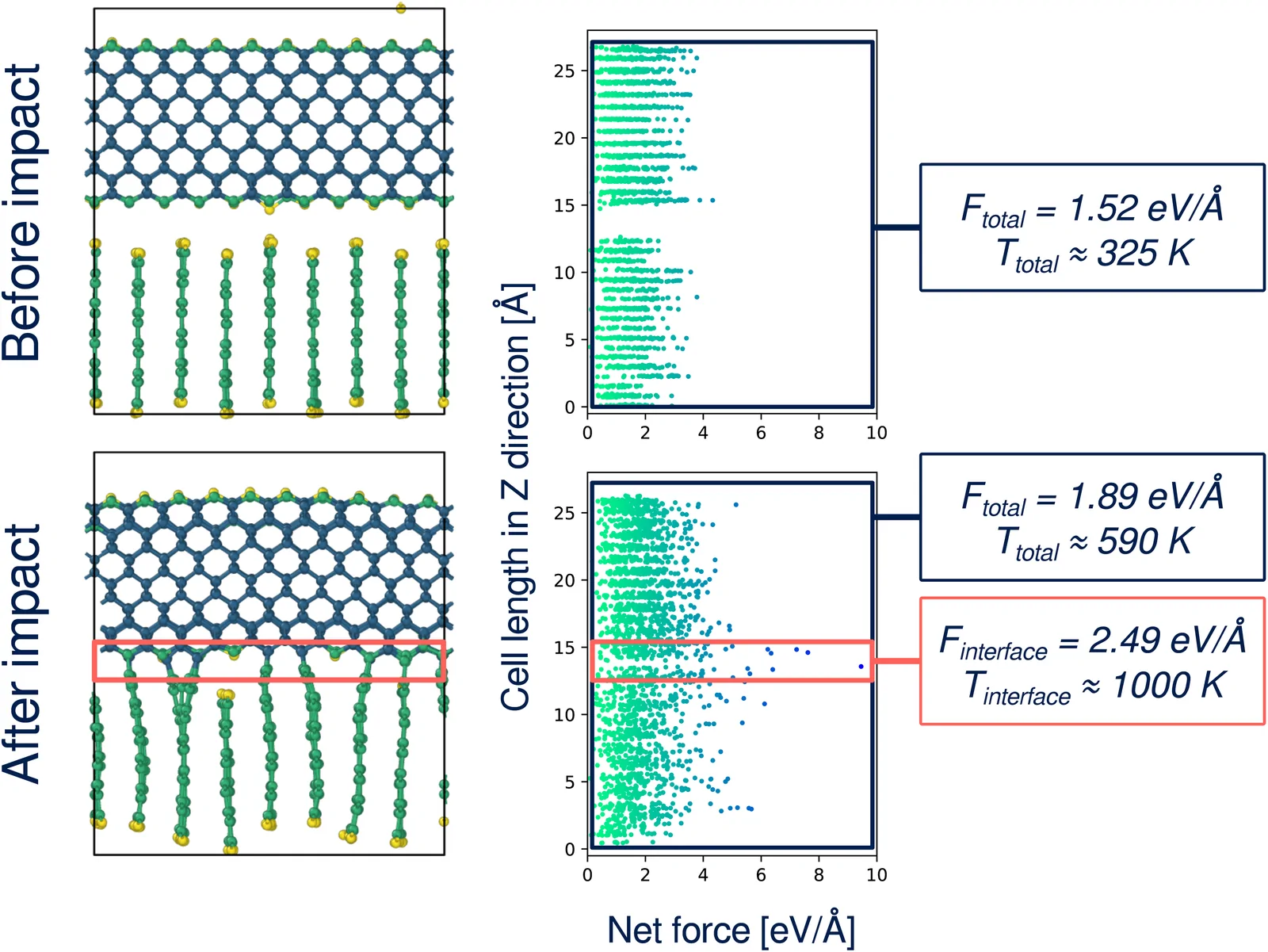
An example of the net forces distribution before and after collision (at 0.1 and 0.7 ps, respectively) with respect to *z* direction of the unit cell (diamond slab with 100 facet and graphitic layers at 90°, 30% compression). Since net force is velocity (*v*), and temperature (*T*) is a measure of kinetic energy of particles, temperature at the interface was estimated from eqn (1). The interface was defined as a region with *z* ∈ 〈12.5, 15.5〉 of the unit cell. Forces were filtered with respect to this region and averaged.

In the simulation, velocity is practically a net force acting on an atom. Fig. 3 shows an example of the distribution of net forces with respect to the length of the cell in the *z* direction before and after impact. Before the collision, the net forces are equally distributed, and the temperature is low. When the impact occurs, there is a visible increase in net forces, at the interface in particular, and also in the temperature. Due to the much greater increase in forces at the interface, it can be assumed that, as expected from the collision event, the interfacial heating occurred and was captured in the simulations.

We can define the collision interfacial temperature (*T*_interface_) in terms of temperature of the whole unit cell (*T*_total_) and a square of average velocity of all particles (*v*_total_) and particles at the collision interface (*v*_interface_):1
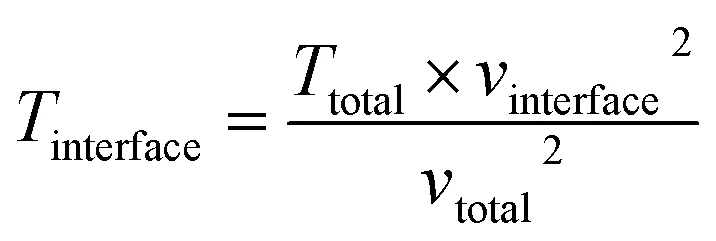
which corresponds to calculating the temperature at the interface only. (Fig. 3) Notably, the obtained value should be treated as an effective non-equilibrium quantity rather than a static one. Consequently, while the temperature of the whole cell was around 590 K, the interfacial temperature was estimated to be around 1000 K. This indicates that the simulations behave physically, displaying localised heating at the interface. It also suggests that C–X bond cleavage (where X is a heteroatom), which is likely to be a limiting factor for graphite–diamond bonding to occur, is potentially achievable under high-speed collision conditions.

**Fig. 4:**
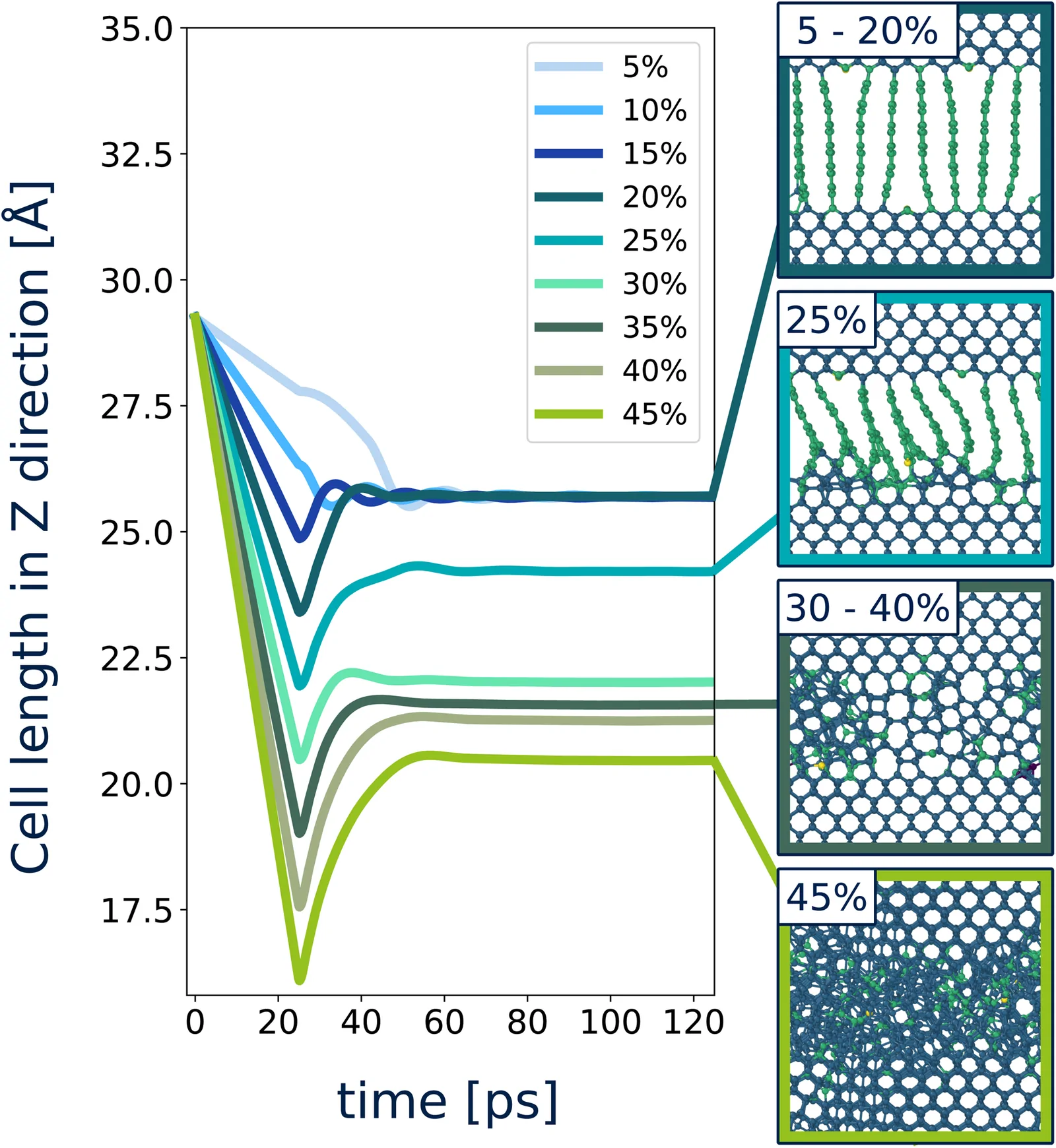
An example of change in cell length in *z* direction of the box over time, under different percentage compressions, along with the final structures after relaxation (diamond slab with 100 facet and graphitic layers at 90°). Substantial changes in length resulted in substantial structural changes.

Having established the existence of surface heating, we can return to Fig. 2. As compression increases, so does the percentage of final sp^3^ atoms. In most cases, at least the first two lowest compressions (5% and 10%) induce no change to the graphitic domain (Diaphite (gra → gra) bars). Upon increasing compression, graphitic domains undergo structural changes, while the diamond domains remain essentially unchanged. First, only a fraction of sp^2^ atoms turn into sp^3^ (Diaphite (gra → gra) + sp^3^ bars). As the compression increases, more sp^2^ carbons become sp^3^, eventually leading to complete transformation of the graphitic domain into the diamond-like structure (Diamond-like (gra → sp^3^ bars). Finally, at the highest compressions, nearly all structures show amorphisation – entirely or partially. The formation of the amorphous phase is not related to the initial placement of the graphitic and diamond domains (a-C (sp^3^ + gra → a-C bars) The diaphite structure seems to be preserved for all the systems showed up to 20% compression, while at 35%, graphite is turned into the diamond-like phase for the most of them. Noteworthy, the increase in the final sp^3^ percentage is not linear with respect to compression. Instead, it stays roughly the same at lower compressions, then increases rapidly. Fig. 4 shows that for nine different compressions, only four distinctly different structures were obtained. The change was indicated by the noticeable difference in the length of the cell in the *z* direction.

**Fig. 5:**
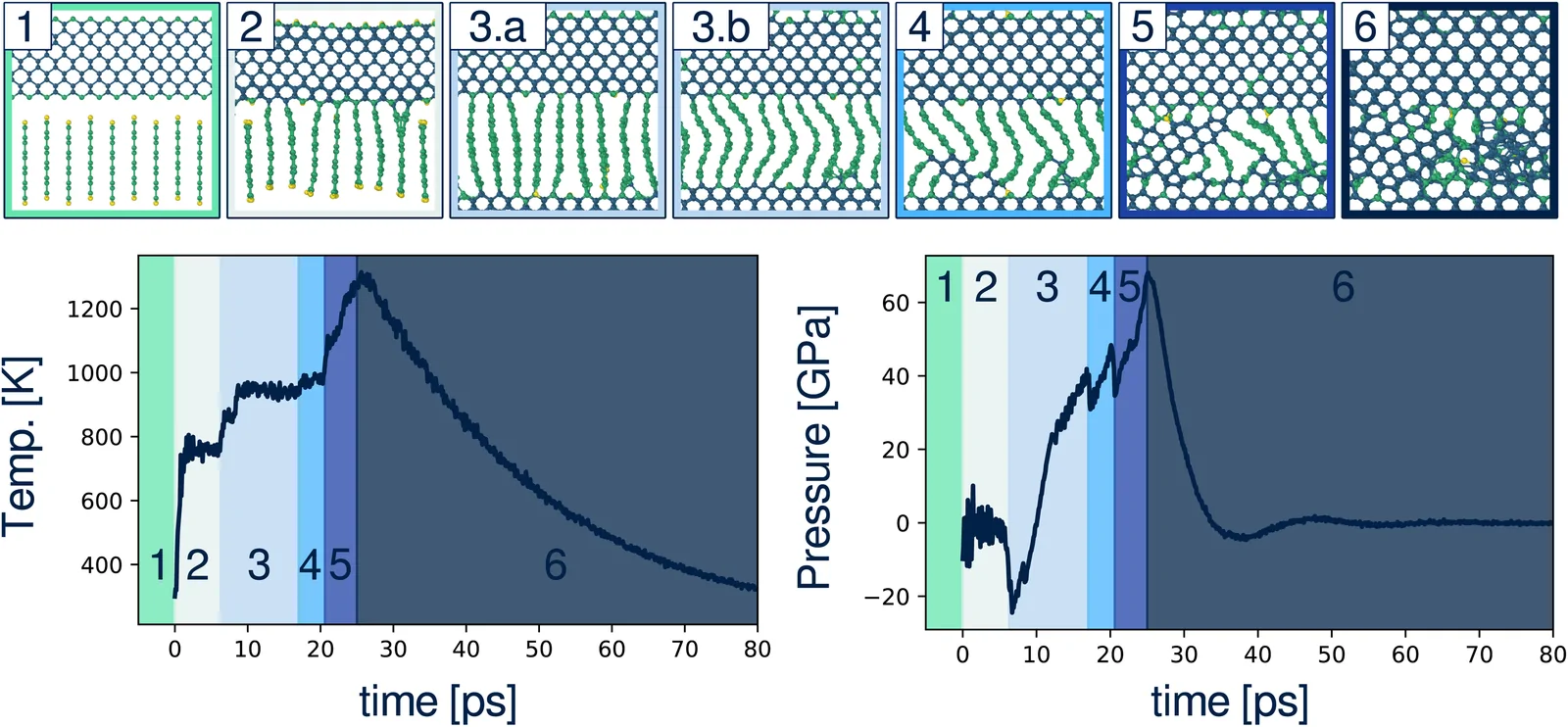
Example of structural changes throughout the simulation and related to them changes in temperature and pressure in *z* direction (diamond slab with 100 facet and graphitic layers at 90°, 30% compression). Increase in temperature indicates bond formation, while drop in pressure suggests transition to more compressible structure.

This behaviour highly resembles a classical spring which can be stretched or compressed and then returns to its relaxed length. This observation is important for speed of collision calculations, as it infers that, rather than a single value that would massively limit viability of the collision-based synthetic route, there is likely to be a range of speeds at which bonding between domains can occur without major changes to the structure. The spring analogy does not end here. Classically, when a spring is loaded beyond its elastic limit, it becomes permanently deformed. An equivalent situation is presented in Fig. 4 for 25% compression. The graphitic layers reached their “elastic limit” and part of them were turned into diamond-like structure, reaching stable configuration. According to Fig. 4, the transformation of the graphitic domains into the diamond-like structures initiates from the grain boundary with the diamond domain. The same phenomenon is observed in Fig. 5, which shows the step-by-step structural changes and how they relate to temperature and pressure.

First, graphitic layers are bent (Fig. 5(3a) and (3b)). Then, the sp^2^ atoms closest to the boundary turn into sp^3^, forming various types of rings or diamond-like structures (Fig. 5(4)). Finally, as the compression increases, the diamond-like motifs propagate (Fig. 5(5)), eventually leading to the complete transformation of the graphitic domain (Fig. 5(6)). This mechanism was observed in all systems, regardless of diamond facet or graphitic plane orientation. Although all structures adopt the same mechanism, some notable differences are worth mentioning. The transition that graphitic slab undergo upon collision with diamond surface is largely determined by orientation of graphitic planes. Parallel graphitic layers (with respect to the direction of the applied pressure), tended to transform to diamond along the 111 facet; alternatively, they form hexagonal diamond. Intermediate orientations (with graphitic planes at 30°, 45° and 60°) change into diamond along the 110 facet. Both of these observations are consistent with the direct graphite to diamond transformation outlined by Xie *et al.*,^9^ where both parallel and bent graphitic layers follow the same growth trends as observed here.

Conversely to other orientations, perpendicular graphitic planes most often propagate along the diamond facet from the colliding slab. This is a result of the unfavourable geometry with respect to the applied pressure. Graphitic planes at this orientation have no preference for a diamond transition along any of the facets, therefore, when forced to form a more compressed phase, they simply adapt to a given template, here that provided by the diamond slab.

These observations are significant in the context of collision-based diaphite synthesis optimisation. As the inner structure of the interconnected domains tracks back to its initial phase, the degree and type of change can be used as an indicator for the need to decrease the speed of collision. The analysis of the post-collision samples could provide insight on the abundance of facets and likelihood of their collision with graphitic planes at specific angles.

Changes in structure can be related to changes in pressure and temperature. As shown in Fig. 5, the sharp increase in temperature is related to the bond formation. The more bonds formed, the greater the increase, as is evident when comparing the change between stages 1 and 2 to the change between stages 3 to 4. Pressure generally increases until the end of the compression phase (corresponding to the first 25 ps). However, there are notable drops in pressure, strongly correlated with the formation of a new, stable structures. Positive pressure translates to the system expansion, while negative pressure relates to the system's tendency to shrink, or to be compressible. This explains why the pressure component in the *z* direction falls dramatically when both graphitic edges are bonded to diamond surfaces – the system reaches a more stable configuration as compared to moving separate domains. As it is further compressed, the graphitic layers bend (Fig. 5(3b)), increasing pressure, as the system pushes to expand. The “elastic limit” is reached, bonding at the interface occurs, decreasing the system's “need” to expand by reaching a more stable state, leading to sudden drop in pressure.

From Fig. 4 and 5, it is evident that compression (which can be expressed as the change in length of the cell in *z* direction), temperature, and pressure are strongly related to the observed structural changes. We would expect a similarly strong correlation between structural change and the speed of the collision. As a result, we propose that, despite the fact that the simulations themselves did not account for collision speed, the speed of collision that would result in a specific structural outcome can be deduced from the observed temperature, pressure, and cell deformation. The next two sections focus on the mathematical derivation of the optimal speed of collision (Section 3.2) and the speed of collision leading to substantial deformation (Section 3.3).

### 3.2 Optimal speed of collision

In the idealised case, the pre-made graphitic domain would simply bond to diamond, but none or a very small fraction would transform into the diamond-like structure. Such cases are represented by the bars in Fig. 2 labelled as Diaphite and whose final sp^3^ percentage stays below 70%. For most systems, it refers to compressions between 5 to 15%, but it varies depending on both the diamond facet and orientation of the graphitic planes, with some being more prone to a phase transition at the interface than the others as discussed in the previous section.

**Fig. 6:**
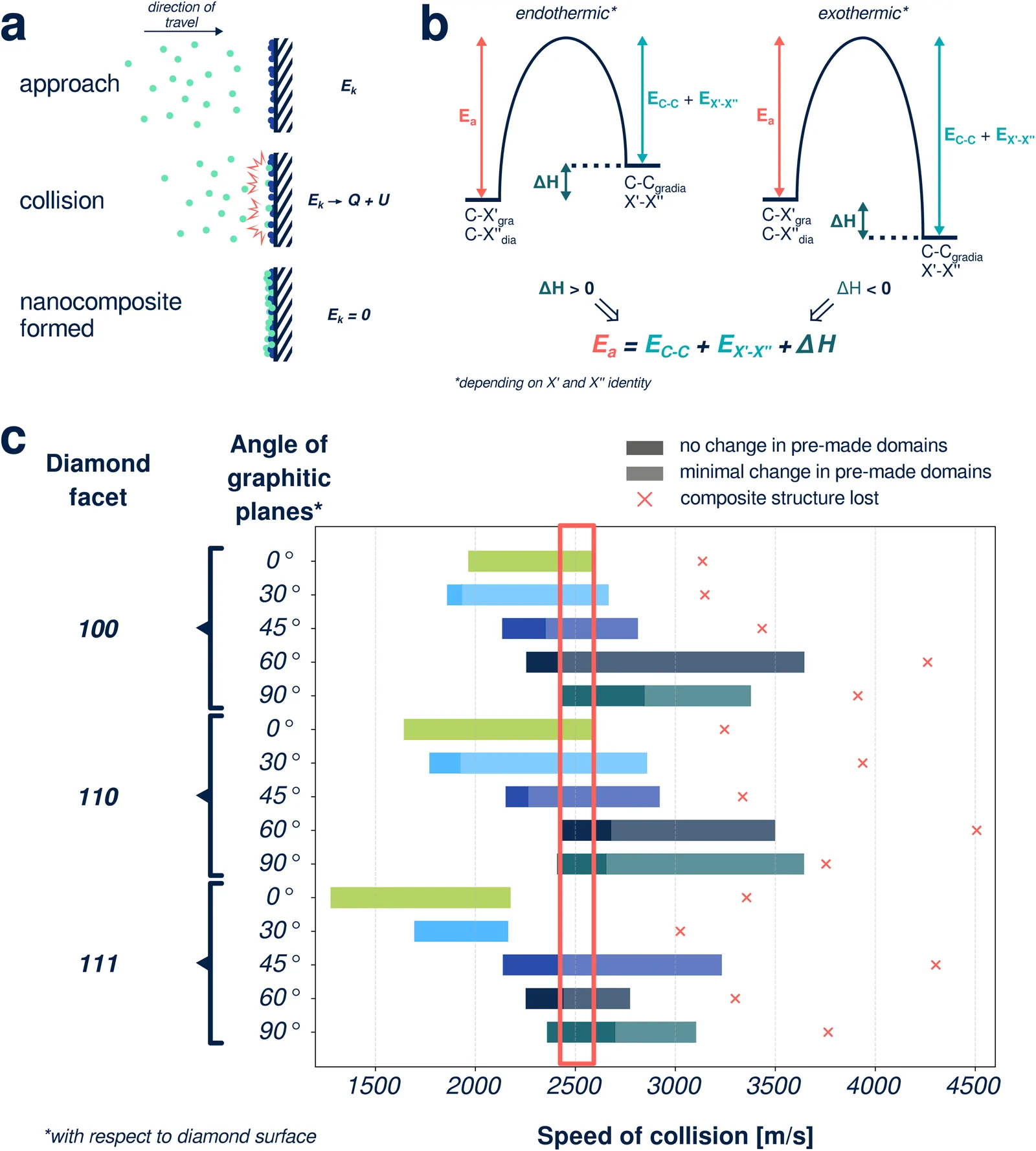
Speed of collision calculations: assumptions and results. (a) Simplified representation of collision based synthesis with one type of particles being fixed on the surface. (b) Relationship between energy of activation (*E*_a_), enthalpy (Δ*H*) and energy of bonds formed (*E*_C–C_, *E*_X′–X″_) for endothermic and exothermic reactions depending on the identity of X′ and X″. (c) Ranges of speed of collisions leading to diaphites with respect to diamond facet and orientation of graphitic planes involved in the collision. Bars correspond to the speed where no change in the structure of pre-made domains is observed (intense colouring) and to the speed of collision which leads to minimal but visible changes to the domains' structure (pale colouring). Speed at which composite structure is lost is marked with an × sign. The rectangle marks an “optimal window” of speeds at which most of the facets and graphite plane orientations are expected to form diaphites either with no or minimal change to the pre-made domains.

The main source of energy in collision-based synthesis is the kinetic energy (*E*_k_) of the particles. Since the particles form bonds, the collision is inelastic, meaning that the energy is converted into heat (*Q*), deformation (*U*), and vibration (*V*). For ease of argument, we assume that none of the kinetic energy is retained, *ergo* particles' velocity after collision is zero. It is the equivalent of having one set of particles fixed on the motionless surface and propelling another set at it (Fig. 6a).

2 *E*_k_ = *Q* + *U* + *V*

Lattice vibrations contribute to the temperature of the system as the phonons are thermalised. Considering the relatively small size of the systems, all phonons should have been thermalised before the end of the compression phase, contributing to the increase in temperature. As a result, eqn (2) can be reduced to:

3 *E*_k_ = *Q* + *U*

*Q* is expected to be related to change in temperature, while *U* to the change in pressure. Starting with *Q*, as the amount of heat absorbed by the material to heat it up by temperature Δ*T* is related to its heat capacity, it can be expressed as follows:

4 *Q* = *C*_s_ × *m*_total_ × Δ*T*

where *C*_s_ is specific heat capacity, *m*_total_ is a sum of the mass of diamond and graphitic domains, and Δ*T* is the difference between final and initial temperature. The term “final” is understood as the averaged temperature reached for the specific structure, similarly to that presented in Fig. 5, snapshots 2, 3, and 4, along with the relevant stages in the temperature against time plot. In the case of lower compressions, leading to bonding only, but no further change in structure, only one temperature increase is observed.

As explained in Section 1, diaphite can be considered as graphite and diamond simply connected by the interface. Following this simplified model, *Q* can be expressed in terms of a linear combination of *C*_s,gra_ and *C*_s,dia_, weighted by their mass.

5 *Q* = (*C*_s,gra_ × *m*_gra_ + *C*_s,dia_ × *m*_dia_) × Δ*T*

where *m*_gra_ + *m*_dia_ = *m*_total_. Naturally, a more realistic description of heat capacity of diaphites is likely to be related to the nature of grain boundaries and size and shape of domains. However, at this stage of the preliminary investigation, such an approximation was deemed sufficient.

As shown in Fig. 5, temperature increases are related to bond formation. The presented approach ignores the potential formation of bonds other than between carbon atoms, so needs relevant corrections to account for them. Real graphitic edges and diamond surfaces can be terminated with heteroatoms, or according to Posligua *et al.*, in case of the graphitic edge, the layers can bond to one another, forming closed-edge graphite.^17^ Nevertheless, bonding of graphite–diamond interface requires bond breaking at the interface and eqn (3) does not account for that. As a results, we need to consider the reaction at the interface. The reaction between graphitic edge and diamond surface, leading to interface formation, can therefore be approximated to:C–X_dia_^1^ + C–X_gra_^2^ → C–C_gra,dia_ + X^1^–X^2^(Δ*H*′),where the subscripts “dia” and “gra” refer to bonds at the diamond surface and graphitic edge respectively, and X^1^ and X^2^ stand for the heteroatoms. The reaction will be different when graphitic planes are at 0° with respect to diamond surface, as the graphitic plane does not possess any heteroatoms. In that case, the reaction can be represented as follow:C–X_dia_^1^ + C–X_gra_^2^ + 6C ≈ C_gra_ → 2C–C_gra,dia_ + 6C–C_gra_ + X^1^–X^2^(Δ*H*″)

Two recombining heteroatoms must be sourced from the diamond surface, hence C–X_dia_^1^ + C–X_gra_^2^. C ≈ C_gra_ stands for bonds within graphitic sheet, ≈ sign denoting their partial double bond character. As the conjugation is effectively broken, leading to the exchange of partial double bonds for single bonds, they were also included in the equation. For the sake of simplicity, we have assumed that both the diamond and graphitic edge are H-terminated, leading to H_2_ formation (X^1^ = X^2^ = H).

The magnitude of the required correction is likely to be linked to the C–H activation energy which, in the presented work, is approximated to the bond dissociation energy (BDE) as high temperatures at the interface (Fig. 3) can facilitate radical formation. In the absence of kinetic data estimation of the activation energy, *E*_a_, is difficult. For our purposes, it can be estimated by using the BDE for the C–H bond in alkenes or by looking at the enthalpy change diagram. While the first approach seems to be simpler and has been employed previously,^26^Fig. 6b and eqn (7) highlight how the second approach appears to offer a better connection to the simulation by incorporating *Q*. Additionally, both the bond dissociation energy of H_2_, BDE_H–H_, and the enthalpy of reaction at the interface, Δ*H* are less elusive than BDE_C–H_ itself.

6 *E*_a_ = *E*_C–C_ + *E*_H–H_ + Δ*H*

7 = *Q* + BDE_H–H_ + Δ*H*

Both *Q* (from eqn (5)) and BDE_H–H_ are terms related to energy released upon bonding and Δ*H* is the difference between the energy used up and gained in the process (Fig. 6b). As a result, the final form of the correction (referred to as *E*_correction_) that needs to be added to eqn (3) is:

8 *E*_correction_ = BDE_H–H_ + Δ*H*

It is worth noticing that Δ*H* can be positive or negative depending on the heteroatoms at the surface/edge which influence the magnitude of the correction (Fig. 6). As the C–H bond has the highest bond dissociation energy among those bonds normally found on diamond surfaces or graphitic edges, assuming H-termination allows us to determine the upper velocity limit. The realistic speed of collision leading to bonding is likely to be lower due to variation in bonds at the graphitic edge and diamond surface. This aspect is further discussed in Section 3.4.

The final term to consider is *U*, deformation energy. In Section 3.1, we noted that Fig. 4 and 5 show that the system displays spring-like behaviour. Classically, the spring deformation energy is expressed as:9
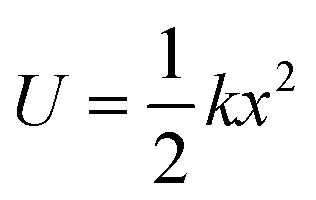
where *k* is a spring constant – a measure of its stiffness – and *x* is a displacement from the equilibrium distance. As the system in question is simply compressed anisotropically in the *z* direction, *x* can be replaced with the appropriate change in length of the cell (Δ*z*). *k* is classically expressed by:10
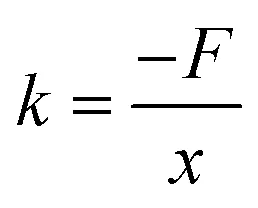
where *F* is force, required to deform a spring by length *x*. The force required to deform the spring-like system presented in this work can be obtained from simple relationship between force and pressure (*p*): 
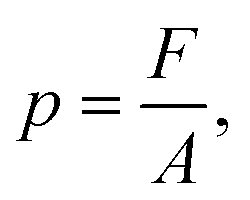
 as the maximum pressure is known and the force is acting over constant area (*A*), being the cross section of the supercell. As a result:11
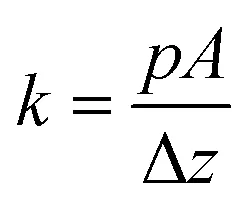
and hence:12
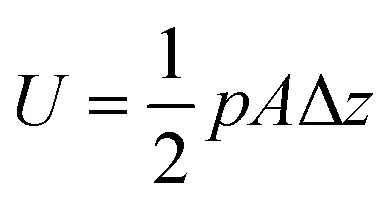


Finally, the kinetic energy is simply:13
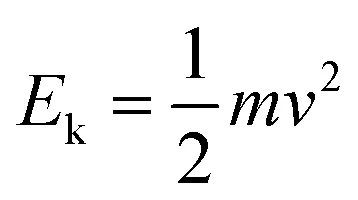
where *m* is the mass and *v* the speed. In our case, *m* is the total mass (*m*_tot_) of the system (*i.e.*, including both types of particles) and *v* is their relative speed. For the case when one type of particles is fixed on the surface, the relative speed is simply the speed of approaching particles.

The substitution of terms in eqn (3) with eqn (5), (12), and (13) and including *E*_correction_ (eqn (8)), leads to the speed of collision (*v*):14
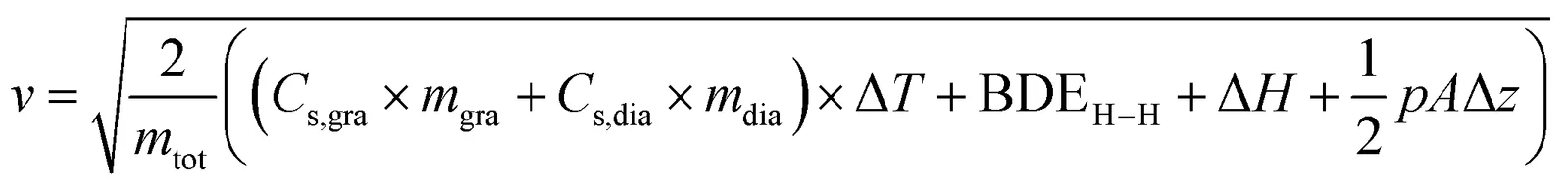


This equation allows us to calculate the speed of collisions that lead to formation of diaphite with no phase transition at the interface (Fig. 6c).

### 3.3 Critical speed of collision

Having determined the optimal speed of collision, another useful term is a critical speed, *i.e.* the speed of collision likely to lead to significant changes in the initial domains. This critical state was defined as a complete transformation of the graphitic domain into a distinct sp^3^ domain, considering the fact that real-life particles are expected to be larger than those examined in the current paper.

To account for the step-wise changes in the structure (see Fig. 5), eqn (14) has to be adjusted. Changes in structure, *i.e.* advancing diamondisation, should influence the heat capacity. As shown in Fig. 5, when the structure stays the same while being compressed without further bonding, the temperature of the systems remains constant. *Q* term can be modified by the addition of the new heat capacity terms, corresponding to the formation of new structures.15
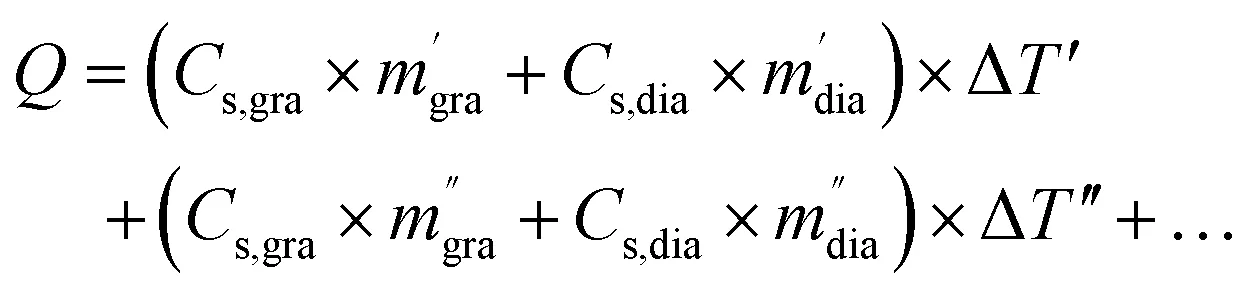
*C*_s,gra_ and *C*_s,dia_ are specific heat capacities of graphite and diamond, *m*_gra_ and *m*_dia_ are masses of graphitic and diamond part of the structure and Δ*T* is the increase of temperature upon formation on a new structure. Superscripts correspond to different stable structures formed during the process. Each stable structure formed should have its term in the equation. The number of additional terms depends on the number of “stairs” visible in the temperature over time plot (Fig. 5).

Another important factor is the change in correction *E*_correction_ (eqn (8)). Usually, the number of heteroatoms at the graphitic edge (or in case of carbon-only systems, number of under coordinated atoms) is smaller than that at the diamond surface. As a result, further transformation of the graphitic layer is likely to involve breaking the remaining C–H bonds at the diamond surface. The reaction would resemble that between parallel graphitic layers and diamond facets, as only the latter is the source of heteroatoms. In this case, correction changes to:

16 *E*_correction_ = BDE_H–H_ + Δ*H*′ + Δ*H*″,

where Δ*H*′ refers to the enthalpy of the edge–surface reaction and Δ*H*′ refers to the plane–surface reaction with the remaining hydrogens. Each should be weighted by mass or number of moles of hydrogen generated. When all of the heteroatoms at the surface form gaseous product phase transition involve carbon atoms only and so the energetics should be reflected directly in temperature changes with no further corrections needed.

The final term of eqn (14) does not change – it still takes maximum pressure (*p*) exerted on the system, over a specific area (cross section, *A*) when compressed anisotropically (by Δ*z*). It is simply the extension of the spring analogy – while the spring-like, elastic behaviour are observed mostly for graphitic layers, each chemical structure, each bond can be treated as spring of a certain stiffness. It is evident from Fig. 4 which shows that regardless of the compression magnitude, the structure expands upon relaxation, which means it retains its elasticity to some degree and the whole compression event can be treated as performed on single spring with averaged behaviour.

An alternative, similarly to eqn (15), would be to account for the formation of different structures. However, such an approach would be in fact double counting of the energy. Elastic deformation energy is converted into heat upon forming new structure and is explicitly included in eqn (15). As a result, to estimate how much energy went into deformation, only the maximum pressure needs to be considered.

The critical velocities are presented in Fig. 6, marked by × sign.

### 3.4 Speed of collision: differences between systems, surface chemistry, size effect, order of magnitude and perceived properties of graphite

We previously defined an optimal collision speed as one that does not induce any structural changes in either domain. While this definition holds for reactions between a graphitic edge and a diamond surface, some degree of phase transition is unavoidable for parallel graphitic planes. In these cases, the terminating graphitic layers must react with the diamond surface, eliminating the heteroatoms passivating it; otherwise, diaphite does not form. In light of this requirement, as well as the poor overlap (Fig. 6c) between optimal speed ranges for most edge–surface configurations, we included additional simulations that allowed for limited structural changes within the graphitic domain while preserving the diaphite structure (pale bars in Fig. 6). These calculations followed the methodology described in Section 3.3 for determining critical speeds. As a result, the accessible collision-speed window for diaphite formation was broadened and now encompasses nearly all systems studied. Although this window does not include the 111 facet combined with graphitic planes at 0° and 30°, the common optimal range remains well below the corresponding critical speeds at which the composite structure is completely lost (Fig. 6). The absence of overlap in these cases may be due to the incremental changes applied to the simulation box compression. This explanation is supported by the unusually large gap—approximately twice that observed in most other systems—between the upper diaphite formation limit and the corresponding critical speed, suggesting that the full diaphite range was not captured.

Excluding the out-of-distribution cases (collision between 111 facet and graphitic planes at 0° and 30°), both optimal and critical speeds generally increase as the orientation of the graphitic planes becomes more perpendicular to the diamond surface. This trend can be attributed to two main factors. Firstly, as the angle at which the graphitic planes collide with the diamond surface approaches 90°, the number of C–C bonds formed per unit area increases (see Fig. 1). As the correction *E*_correction_ introduced in eqn (8) and (16), depends on the number of bonds formed, a higher density of heteroatoms at the graphitic edge per unit area requires a greater collision speed (eqn (2)).

The second contributor to the increase in collision speed is the pressure-related component, which is best captured by the trend in system stiffness expressed by the parameter *k* within the spring analogy framework. Average values of *k* increase systematically with plane orientation, taking values of 80, 124, 210, 296, and 425 N m^−1^ for angles of 0°, 30°, 45°, 60°, and 90°, respectively. A stiffer system can accommodate greater energy through elastic deformation without undergoing structural change. While the increased stiffness of graphitic layers under in-plane compression is intuitively expected, it is quantitatively confirmed here by the observed trend in *k*. This agreement indicates that the spring analogy is not merely a convenient approximation, but reflects the underlying atomic-scale response of the system.

It is important to note that presented speed of collisions are unlikely to be universal due to the differences in the chemistry of real-life surfaces and edges mentioned briefly in Section 3.2. Due to the high energy of a C–H bond, and most importantly, due to the endothermic nature of the reaction, the speeds of collisions are driven up. Both diamond surfaces and graphitic edges can contain other atoms that may substantially change the chemistry and the energetics of the reaction upon bonding. For example, if some H atoms are replaced with OH groups, the product of the recombination reaction will be H_2_O, rather than H_2_, with exothermic effect of Δ*H* ≈ –16 kJ compared to Δ*H* ≈ 82 kJ. This can not only lower the BDE term but also physically increase the temperature of the surface facilitating further bond breaking events, effectively lowering the required speed of collision. Another example of structural change that could impact the necessary speed of collision are closed edges at the graphitic edge. As this reaction requires only half the amount of C–H bonds to break, compared to the example considered in detail in this paper, the BDE term is again reduced. The realistic speed of collision is therefore highly dependent on the surface chemistry, and the computationally efficient models must rely on some sort of simplification. Even so, the values calculated here represent a useful set of results as they are likely to approach the upper limit of both optimal and critical speed due to the high energy of C–H bonds.

Another important factor that might substantially influence realistic speeds of collisions is the finite size effect. The current model assumes the collision occur between infinitely flat surfaces and, due to periodic boundary conditions in all directions, the system behaves somewhat similar to the bulk material. It is therefore the most accurate for sufficiently large particles, likely in the size regime of micrometres rather than nanometres. The precise size effect is hard to estimate based on the results alone but in principle, it can be influenced by two effects. First, as the proportion of atoms at the interface with respect to bulk increases, the surface reactivity increases which might potentially lower the C–X activation barrier (X being a group passivating the particle) and effectively lower the necessary speed of collision. On the contrary, decreasing the size of a particle, decreases the cohesive energy within the particle itself which is especially important in the case of graphite when approaching graphene regime. In such case, substantial deformation could potentially be observed at much lower speeds, likely destroying the particle integrity and not leading to diaphite at all.

In general, both optimal and critical speed calculations carry an inherent degree of uncertainty and may therefore appear unconvincing at first, particularly in the context of collision-based synthesis. To address this concern, three key points must be considered. The first point concerns the wide ranges of collision speeds, which can vary substantially and, in some cases, differ by more than 500 m s^−1^ (see Fig. 6c). Crucially, however, all systems—regardless of diamond facet or graphitic plane orientation—form diaphite at speeds below the lowest critical value. In principle, as long as this lowest critical speed is not exceeded, none of the initially prepared domains should undergo complete structural transformation.

The second point concerns the magnitude of the critical speeds. For the limiting cases of 0° and 90° graphitic plane orientations, the average critical speeds are 3246 m s^−1^ (standard deviation: 110 m s^−1^) and 3810 m s^−1^ (standard deviation: 89 m s^−1^), respectively. These values are in good agreement with the work of Chen *et al.*,^27^ who estimated the shock-wave velocities associated with the first observable transition from graphite to diamond to be approximately 3.5 km s^−1^ for the [0001]_G_ orientation and 4.0 km s^−1^ for [101̄0]_G_. This correspondence is notable given the relatively simple nature of the present estimates. The slightly lower velocities observed in diamond–graphite particle collisions may arise from a templating effect of the diamond domain following initial bonding. Although the graphitic layers do not necessarily adopt the same diamond facet as the slab, the presence of the diamond phase can still reduce the energetic barrier for the sp^2^-to-sp^3^ transition. Complementary evidence is provided by the study of Barbaro *et al.*,^28^ who showed that graphite begins to transform into diamond under shock pressures exceeding 15 GPa, and most commonly above 30 GPa. In the present work, pressures associated with critical cases—where the composite structure was lost—range from 25 to 72 GPa, fully consistent with the expected regime for graphite-to-diamond transformation.

Finally, there remains the question of the apparent cognitive dissonance between the calculated results and the conventional perception of graphite. Graphite is commonly encountered either as an inert component of a pencil or as a lubricant in the automotive industry, applications that rely on its softness and ease of structural disintegration. At first glance, the survival of graphitic particles during collisions at such high velocities – without immediate fragmentation or amorphisation – may therefore appear counter-intuitive. However, this apparent contradiction is resolved when considering the fundamentally different material response under shock loading compared to static or quasi-static conditions. Indeed, amorphisation is observed in the simulations, as expected, but only at substantially higher compressions and collision speeds. Moreover, structural changes consistently initiate within the graphitic domain, while the diamond domain remains largely unaffected, in agreement with theoretical expectations. These observations indicate that the system behaves in a physically consistent manner, even if such behaviour cannot be readily inferred from macroscopic experience. Although particle size is expected to play an important role—a factor that is difficult to assess given the use of periodic models—the present results should not be regarded as disqualifying in view of the unique nature of the shock conditions considered.

### 3.5 Available synthesis techniques

With the theoretical framework established, it is now useful to place these results in a practical experimental context. Although collision-based synthesis has not previously been proposed for diaphites, it is well established in materials science and chemistry. In a broad sense, all chemical reactions involve collisions; what distinguishes the present case is the requirement for a sufficiently high collision speed combined with a very short interaction time, such that the integrity and phase of the pre-formed domains are preserved. Several existing techniques may therefore be considered as potential experimental analogues.

Note that the presented model serves solely as an approximation for the collision of real-life particles. Under the employed periodic boundary conditions, it is effectively an anisotropic collision between two, infinite and flat surfaces. Consequently, it neglects aspects such as the particles' shape and curvature, as well as their rotation, translation or lateral compression as modes of redirecting energy of an impact.

First, since the particle surface is complex in nature, experimental tuning is likely to be challenging. Same conditions might result in good bonding for some combinations and in the complete lack of it for the others. The structure can also be expected to display voids or unbound surfaces as a result of limited surface-to-surface contact. Second, upon impact, finite particles could simply slip instead of forming bonds. The shape of the particles is also likely to change and this could in principle impact the properties of the resulting composite. However, these should be mitigated when collisions occur in confined spaces in which movements and deformations of particles are restricted from the start.

In light of these, the techniques mentioned below should not be treated as definite, but rather as hypothetically useful methods that require a careful evaluation and planning in the context of the physical particle behaviour.

The method most closely resembling the approximation adopted in Section 3.2 and Fig. 6a is thermal spraying. This technique relies on high-pressure gas streams to accelerate particles toward a substrate, producing collision velocities in the range of approximately 250–1250 m s^−1^.^29^ These speeds are roughly a factor of two lower than the calculated optimal ranges. However, it is important to emphasise that the present calculations represent an upper theoretical limit, assuming complete saturation of both the graphitic edge and diamond surface with hydrogen – a condition that is rather unlikely in the experimental setting. Consequently, the collision velocities accessible in thermal spraying may in practice be sufficient to promote diaphite formation. Additional surface preparation strategies could further lower the required speeds. For example, functionalisation of the diamond surface with –OH groups would reduce the relevant bond dissociation energies, since tabulated BDE_C–H_ values are generally higher than the corresponding BDE_C–OH_.^24^

Despite these advantages, thermal spraying is inherently limited to coating applications. For the synthesis of bulk diaphites, alternative consolidation techniques must therefore be considered, such as spark plasma sintering or explosive powder compaction. Spark plasma sintering produces dense solids from powders by passing a pulsed direct current through the sample while simultaneously applying pressure.^30,31^ This process generates localised heating at particle interfaces and promotes rapid densification, typically on timescales of only a few minutes. Although spark plasma sintering does not explicitly rely on shock or high-velocity collisions, the underlying mechanism of diaphite formation would be expected to follow the pathway outlined in this work: localised bond breaking and formation at interfaces, followed by pressurization that ensures intimate contact between domains. Parameters such as particle size, applied current density, and processing time would require careful optimization. None of these can be directly related to calculations done in this work. While concerns regarding huge differences in electrical conductivity and hardness may arise, it is certainly a route worth considering.

Explosive powder compaction offers a more direct analogue to collision-based synthesis, as it uses shock waves generated by detonation to accelerate and compact particles within a confined volume. Depending on the explosive used, detonation velocities can range from approximately 1700 to 8400 m s^−1^,^32^ suggesting that this technique could, in principle, access the collision regimes required for diaphite formation. Studies on the formation of nanocrystalline solids *via* explosive powder compaction have demonstrated that nanoscale structural features can be preserved during consolidation,^33^ an important consideration for diaphite synthesis. It should be noted, however, that the calculated values presented in Fig. 6 correspond to relative particle velocities, which must be carefully accounted for in experimental design. Potential drawbacks of this method include significant temperature increases accompanying detonation, which may enhance atomic mobility at interfaces beyond the desired level, as well as inherent safety concerns.

Finally, high-energy ball milling represents a comparatively simple and widely accessible technique, particularly familiar within the chemistry community. This method does involve particle collisions at high operational speeds; however, it also relies heavily on grinding and abrasion.^34^ While diamond particles are unlikely to be significantly affected by grinding, graphitic domains may readily degrade into smaller flakes if collision energies are insufficient to induce true shock conditions. Moreover, the outcome is highly sensitive to operational parameters such as milling speed and duration. Although short processing times are intuitively desirable to limit excessive thermal input and prevent amorphisation, establishing a direct correspondence between the calculated collision speeds in this work and the effective dynamics within a ball mill is not straightforward. The principal concern remains the potential absence of a true shock component, which may lead to degradation of the pre-formed graphitic domains rather than controlled interfacial transformation. Additional challenges include possible damage to milling equipment due to the abrasive nature of diamond. Successful application of high-energy ball milling to diaphite synthesis would therefore require substantial process optimisation or dedicated engineering solutions, such as stepwise substrate addition or the use of milling components designed to promote collisions rather than grinding. If achieved, however, this approach could enable the synthesis of powdered diaphite composites, in contrast to the bulk or coating formats associated with the methods discussed above.

## 4 Conclusions

The present work supports the hypothesis that diaphites could be synthesised from pre-formed diamond and graphitic domains by subjecting them to shock conditions, specifically high-speed collisions. Upon impact, localised heating at the interface should promote bond breaking and bond formation, leading to the emergence of a composite structure. At higher collision energies, the graphitic domain undergoes deformation, exhibiting harmonic spring-like behaviour. When sufficiently deformed, it forms bonds starting at the interface with the diamond domain and can ultimately transform into diamond.

By applying basic physical principles, we derived expressions for estimating relative collision speeds and related them directly to the outcomes of molecular dynamics simulations. Collision velocities leading to complete structural transformation of the domains show good agreement with literature values reported for graphite deformation under shock conditions.^27,28^ Under the assumption of hydrogen-terminated graphitic and diamond surfaces, the optimal relative collision speed was estimated to be approximately 2500 m s^−1^. Appropriate surface functionalisation is expected to further reduce this requirement. Among existing synthesis techniques, thermal spraying, spark plasma sintering, and explosive powder compaction emerge as the most promising candidates, although targeted experimental validation is required to assess their feasibility.

Based on the presented results, we suspect that a collision- or shock-based synthetic approach may offer advantages over conventional high-pressure, high-temperature methods by potentially enabling greater control over the relative proportions of graphitic and diamond components. This finding offers a perspective shift in diaphite formation – moving it away from strict phase transformation pathways toward controlled interfacial assembly of pre-existing domains. We hope that the present work will stimulate experimental efforts in the synthesis of novel carbon polymorphs and composite materials.

## Author contributions

Zuzanna Malinowska-Trzmielak: conceptualisation, methodology, validation, formal analysis, investigation, data curation, writing – original draft, visualization; Nicole Grobert: conceptualisation, supervision, funding acquisition; Mark Wilson: conceptualisation, resources, data curation, writing – review and editing, supervision, funding acquisition.

## Conflicts of interest

There are no conflicts to declare.

## Supplementary Material

RA-016-D6RA04047K-s001

## Data Availability

Data for this article, including LAMMPS input files and MD trajectories, are available at ZENODO at https://doi.org/10.5281/zenodo.19907287. Supplementary information (SI): a decision tree for simulation outcome classification process. See DOI: https://doi.org/10.1039/d6ra04047k.
