# Supplementary material for: Collision-based synthesis of diamond–graphite nanocomposites: computational investigation
Source: RSC Adv. 2026 Jul 20. Online ahead of print. doi: 10.1039/d6ra04047k (PMC13383846; doi:10.1039/d6ra04047k)
Supplement: RA-OLF-D6RA04047K-s001 [file RA-OLF-D6RA04047K-s001.pdf]

Supplementary Information (SI) for RSC Advances.
This journal is © The Royal Society of Chemistry 2026

Supplementary Information: Collision-based synthesis of diamond-graphite nanocomposites: computational investigation

Zuzanna Malinowska-Trzmielak, Nicole Grobert, and Mark Wilson

1 Simulation outcome assignment: decision tree

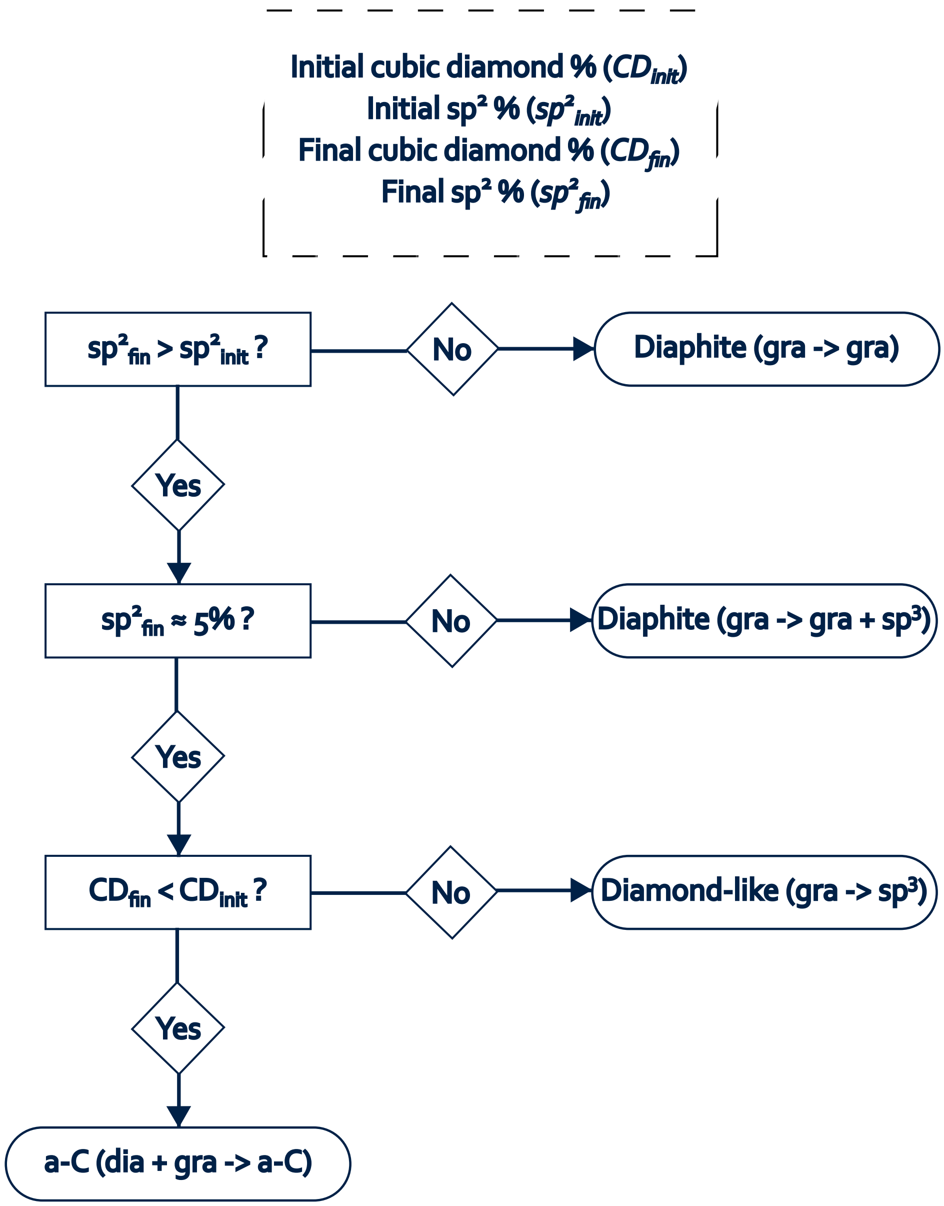

Figure S1: Decision tree for Simulation outcome assignment based on comparison between initial and final percentages of cubic diamond and sp^2. Values were obtained from OVITO[1] using its build-in functions: coordination analysis with single-bond cut-off set to 1.7 Å and diamond structure identification as implemented by E. Maras *et al.*[2]

References

[1] A. Stukowski, *Modelling Simul. Mater. Sci. Eng.*, 2009, **18**, 015012.

[2] E. Maras, O. Trushin, A. Stukowski, T. Ala-Nissila and H. Jónsson, *Comput. Phys. Commun.*, 2016, **205**, 13–21.
